# Comparative genomic analysis of the *Hafnia* genus reveals an explicit evolutionary relationship between the species *alvei* and *paralvei* and provides insights into pathogenicity

**DOI:** 10.1186/s12864-019-6123-1

**Published:** 2019-10-23

**Authors:** Zhiqiu Yin, Chao Yuan, Yuhui Du, Pan Yang, Chengqian Qian, Yi Wei, Si Zhang, Di Huang, Bin Liu

**Affiliations:** 1 Key Laboratory of Molecular Microbiology and Technology of the Ministry of Education, TEDA College, Nankai University, Tianjin, People’s Republic of China; 2 TEDA institute of Biological Sciences and Biotechnology, Nankai University, Tianjin, People’s Republic of China; 3 Tianjin Key Laboratory of Microbial Functional Genomeics, TEDA college, Nankai university, Tianjin, People’s Republic of China

**Keywords:** *Hafnia*, Comparative genomics, Macromolecular secretion system, Virulence factors, Antibiotic resistance

## Abstract

**Background:**

The *Hafnia* genus is an opportunistic pathogen that has been implicated in both nosocomial and community-acquired infections. Although *Hafnia* is fairly often isolated from clinical material, its taxonomy has remained an unsolved riddle, and the involvement and importance of *Hafnia* in human disease is also uncertain. Here, we used comparative genomic analysis to define the taxonomy of *Hafnia*, identify species-specific genes that may be the result of ecological and pathogenic specialization, and reveal virulence-related genetic profiles that may contribute to pathogenesis.

**Results:**

One complete genome sequence and 19 draft genome sequences for *Hafnia* strains were generated and combined with 27 publicly available genomes. We provided high-resolution typing methods by constructing phylogeny and population structure based on single-copy core genes in combination with whole genome average nucleotide identity to identify two distant *Hafnia* species (*alvei* and *paralvei*) and one mislabeled strain. The open pan-genome and the presence of numerous mobile genetic elements reveal that *Hafnia* has undergone massive gene rearrangements. Presence of species-specific core genomes associated with metabolism and transport suggests the putative niche differentiation between *alvei* and *paralvei*. We also identified possession of diverse virulence-related profiles in both *Hafnia* species., including the macromolecular secretion system, virulence, and antimicrobial resistance. In the macromolecular system, T1SS, Flagellum 1, Tad pilus and T6SS-1 were conserved in *Hafnia*, whereas T4SS, T5SS, and other T6SSs exhibited the evolution of diversity. The virulence factors in *Hafnia* are related to adherence, toxin, iron uptake, stress adaptation, and efflux pump. The identified resistance genes are associated with aminoglycoside, beta-lactam, bacitracin, cationic antimicrobial peptide, fluoroquinolone, and rifampin. These virulence-related profiles identified at the genomic level provide insights into *Hafnia* pathogenesis and the differentiation between *alvei* and *paralvei*.

**Conclusions:**

Our research using core genome phylogeny and comparative genomics analysis of a larger collection of strains provides a comprehensive view of the taxonomy and species-specific traits between *Hafnia* species. Deciphering the genome of *Hafnia* strains possessing a reservoir of macromolecular secretion systems, virulence factors, and resistance genes related to pathogenicity may provide insights into addressing its numerous infections and devising strategies to combat the pathogen.

## Background

*Hafnia* is a common genus of gram-negative bacteria found in the environment on plants and foods as well as in the gastrointestinal tracts of mammals. This genus belongs to the *Enterobacteriaceae* family and includes two known species (*alvei* and *paralvei*) [1]. Since its discovery more than 50 years ago, very little has been determined about variations among *Hafnia* species. Previous investigations of the taxonomy of *Hafnia* were based on 16S rRNA, DNA-DNA hybridizations and multilocus enzyme electrophoresis [1, 2]. In addition, many traditional phenotypic characteristics have failed to distinguish these two species from each other [1, 3]. The low cost of next-generation sequencing has generated an upsurge in microbial genome sequences that has allowed researchers to characterize microorganisms using genome data. Therefore, whole-genome sequencing data provide the opportunity to re-evaluate current *Hafnia* species definitions and establish an accurate species definition for the taxonomically challenging genus *Hafnia*.

There are no comprehensive genome-wide analyses of the taxonomy of the *Hafnia* genus. To better understand the phylogenetic relationship and genomic distances that distinguish distinct species, we generated genome sequence data for 20 *Hafnia* strains with different O-antigens and performed phylogeny, population genetic structure and whole genome average nucleotide identity analyses with other publicly available *Hafnia* sequences. To expand the genomic perspective with regard to inter-species diversity and differences, we further constructed core and pan-genome analyses and characterized the species-specific core genome that provides insights into the divergence and niches differentiation between *alvei* and *paralvei*.

Members of the *Hafnia* genus are commonly isolated from the gastrointestinal tract of humans and animals and from foods [4]. *Hafnia,* as opportunistic pathogens, may cause acute gastroenteritis [2]. Notably, it has more commonly been reported to cause severe extra-intestinal disease, including bactereamia and respiratory tract infections, primarily in immunocompromised patients, patients with penetrating soft tissue injury, and organ transplant patients [5, 6]. Unfortunately, very little is known about these strains in regard to their role as pathogens. Benefiting from the increasing feasibility of next-generation sequencing and the advent of bioinformatics tools during recent years, we focused on analysing and defining the virulence-related genetic profiles of* Hafnia* within a large number of genomes to reveal its pathogenic potential.

The use of whole genome sequences has been regarded as a promising avenue for taxonomic and phylogenetic studies of *Hafnia* and for virulence and resistance genotypic profiles of species *alvei* and *paralvei*. In the present study, we generated one complete genome sequence and 19 draft genome sequences for *Hafnia*. These sequences were supplemented by 27 additional genomes of *Hafnia* strains available in public databases. The implementation of our study is divided into three parts: (i) we perform phylogenetic and population structure analysis based on single-copy core genes in combination with average nucleotide identity to elucidate the taxonomy of *Hafnia*; (ii) core and pan-genome analysis of *alvei* and *paralvei* define the species-specific core genome and reveal the inter-species genomic difference, thus expanding our understanding of divergent evolution and adaptation to diverse niches and providing a simple and fast approach for identifying distinct species of *Hafnia*; and (iii) we identify the profiles related to macromolecular secretion systems, virulence, and antimicrobial resistance. The identification of these genomic features will provide further insight into the evolution and pathogenic potential of *Hafnia*.

## Results

### Whole-genome based phylogeny, population genetic analysis, and average nucleotide identity provided a high-resolution taxonomy

**Fig. 1:**
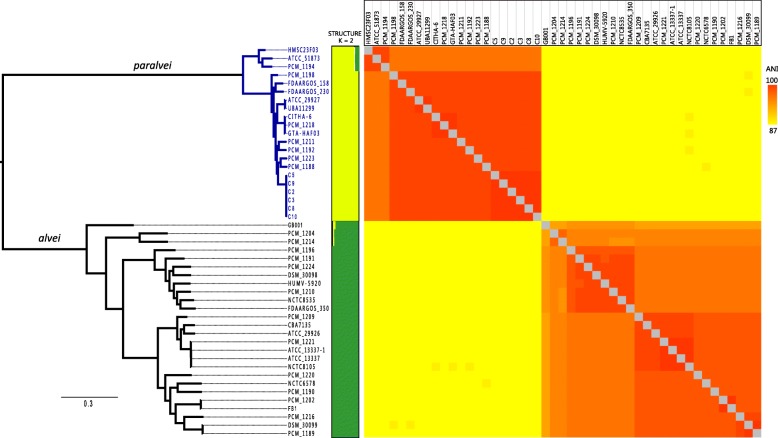
Phylogenetic analysis and whole genome nucleotide identity. A maximum likelihood tree was constructed using PhyML based on 2045 single-copy core genes shared by 47 *Hafnia* strains. The strain names of 21 *paralvei* and 26 *alvei* are indicated in green and black, respectively. Next to the tree, coloured blocks indicate the structure clusters. The colours in the heatmap represent pairwise ANI values, with a gradient from yellow (low identity) to red (high identity)

To assess phylogenetic relationship of the *Hafnia* genus, a phylogenetic tree was constructed using the concatenated nucleotide sequence of 2045 core genes (Additional file 2: Table S2) from 20 newly sequenced and 27 publicly available *Hafnia* strains. The core genome tree generated a reliable delineation of phylogenetic relationships across the *Hafnia* genus. According to our core genome tree, the 47 strains were divided into two phylogenetic lineages (Fig. 1); one lineage contained 26 strains (designated *alvei*), while the other lineage included 21 strains (designated *paralvei*). The lineages *alvei* and *paralvei* formed distinct, extremely tight clusters in separate clades from each other, suggesting that the genetic differentiation between the genomes of *alvei* and *paralvei* strains occurred during adaptation to different niches, and co-evolution with their hosts, in which other microbes could be an important driving factor. To further explore the genomic similarities among strains, we complemented our phylogeny with genetic population structure analysis using the program STRUCTURE [7]. As shown in Fig. 1, the strains generally clustered into two structure clusters in a pattern matching that observed in the phylogenetic analysis. Our phylogenetic analysis and population structure exhibited a reliable delineation of genetic relationships between the species *alvei* and *paralvei*. In addition, we identified a mislabeled strain, ATCC 51873, which was previously classified as the species *alvei* and should be corrected to *paralvei*.

The average nucleotide identity (ANI) value was applied to delineate species and was calculated to estimate the genetic distance between strains at the genomic level [8]. Here we calculated the pairwise ANI values of 47 strains to examine the inter-species genetic relatedness within the *Hafnia* genus. Based on the ANI results, the strains were clearly divided into two groups, which was consistent with the phylogenetic analysis results. The ANI values for *alvei* and *paralvei* were above 96 and 93% (Additional file 3: Table S3), respectively. It is worth noting that the ANI values between *alvei* and *paralvei* were approximately 87%, below the recommended 95% threshold value for species circumscription [8], illustrating the prominent genetic distance between these two species.

### Characterizing the core and pan-genomes

**Fig. 2:**
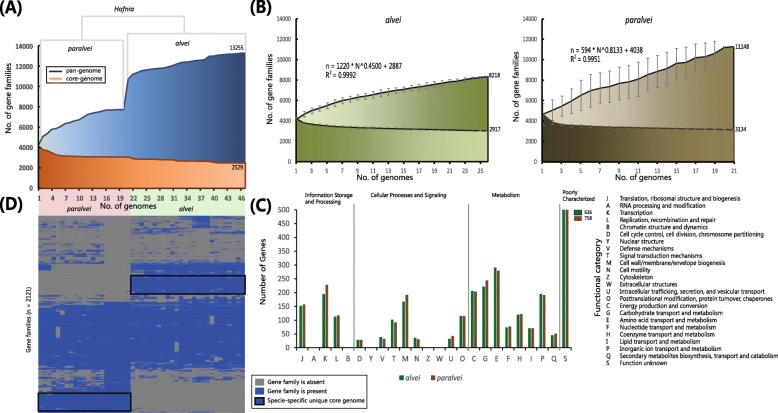
Core and pan-genome analysis of *Hafnia*. **a** The *Hafnia* core and pan-genome were constructed for 47 genome sequences of *Hafnia*, representing 2 species: 21 *paralvei* and 26 *alvei*. The genomes are in the same order as in the core genome tree (Fig. 1). **b** Core and pan-genome curves for genome sequences of *alvei* and *paralvei* show the downward trend of the core gene families and the upward trend of the pan gene families with the increasing number of genomes. We show the average and standard deviation of each value. The deduced mathematical function of pan-genome curves was reported. **c** Distribution of functional catalogues of *alvei* and *paralvei*. **d** Cluster map of the accessory genome of *alvei* and *paralvei*. The gene families that are unique to a species and conserved across most strains in that species are framed in black

To assess the genetic diversity, we constructed the core (genes shared among all 47 strains) and pan (all genes found across all 47 strains) genome curves of the *Hafnia* genus (Fig. 2a). In our pan-genome of *Hafnia*, 13,255 gene families were identified across 47 genomes, of which 2529 constitute the core genome. The pan genome curve is noticeably shaped by the number of novel gene additions with each additional genome. Conversely, a continuous decline in the core genome curve was observed for the novel additional genome. Interestingly, the pan-genome curve was strongly affected by the addition of a large number of novel gene families in additional *alvei* genomes (Fig. 2a), suggesting that there are substantial differences in gene content between *alvei* and *paralvei*.

Having determined the genomic differences, we characterized the core and pan-genomes of *alvei* and *paralvei* separately (Fig. 2b). The pan-genomes of both species show a clear linear upward trend in agreement with the Heap’s law pan-genome model [9], and a robust fit to the data of both species was obtained with an increasing power law with positive exponents of *γ* = 0.4500 (*alvei*) and 0.8133 (*paralvei*). (Fig. 2b). The exponent *γ* > 0 indicates an open pan genome species [9]. Additionally, we found that the *paralvei* pan-genome is approximately 3000 genes larger than the *alvei* pan-genome (11,148 and 8218, respectively), suggesting that strains of this species have more frequent genetic exchange events and a large source of gene content.

Compared to the pan-genome curves, the number of gene families decreased sharply with additional genomes, reaching minimum values of 3134 and 2917 for *alvei* and *paralvei*, respectively. We further used the Cluster of Orthologous Group (COG) assignments to determine the functional categories of the core gene families of *alvei* and *paralvei*. The core gene families of both species were unevenly distributed across the functional categories (Fig. 2c). Larger proportions of the core gene families of both *alvei* and *paralvei* were involved in the transcription (category K: 6.8 and 7.5%), energy production (category C: 7.3 and 6.7%), transport and metabolism of carbohydrates, amino acids and inorganic ion (categories G: 7.8 and 8.0%, E: 10.2 and 9.2%, and P: 6.8 and 6.3%). It is notable that most of the core gene families in both *alvei* and *paralvei* play important roles in maintaining growth and reproduction.

### Species-specific core genomes revealed the extent of divergence between *alvei* and *paralvei*

**Fig. 3:**
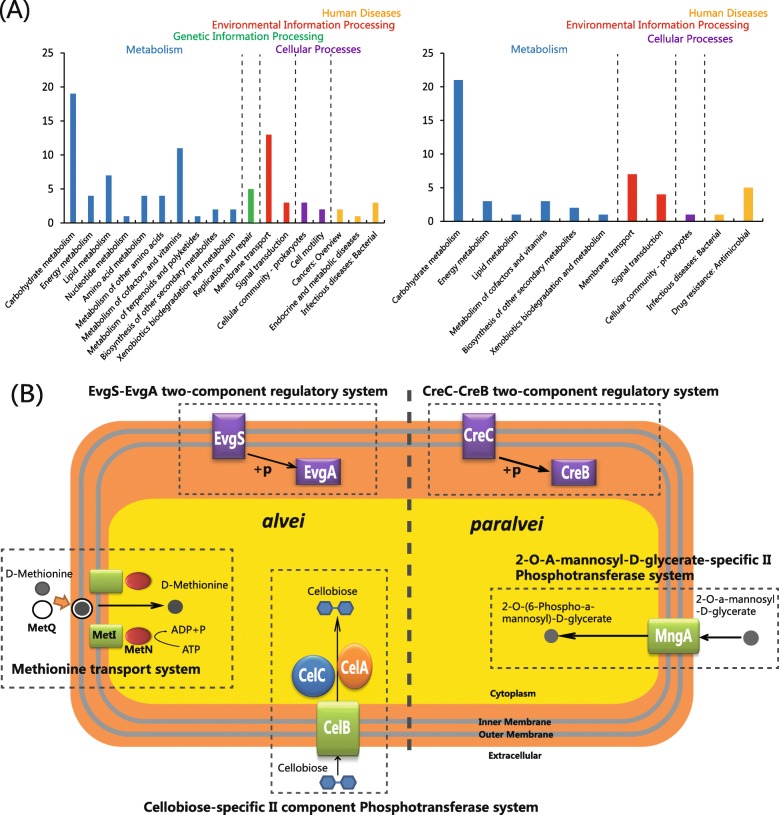
Functional enrichment of the species-specific core genomes after KEGG annotation. **a** The detailed enrichment results of the species-specific core genomes after KEGG annotation. **b** The complete pathway modules in the *alvei* and *paralvei*-specific core genome

The species-specific pan-genome content reveals that these are an underlying profiles of gene families that are conserved among strains of a species, some of which are unique to this species. To identify the species-specific gene families, we constructed the accessory genome by subtracting the core genome and low frequency genes (< 10 examples) from the pan-genome. As shown in Fig. 2d, the cluster map of the *Hafnia* accessory genome demonstrates that each serovar is differentiated by a set of conserved gene families (framed in black, Fig. 2d). A total of 213 and 183 gene families were identified as part of the *alvei* and *paralvei*-specific core genomes (Additional file 4: Table S4), respectively. Bared on KEGG annotation, the functional categories “carbohydrate metabolism”, “lipid metabolism”, “metabolism of cofactors and vitamins” and “membrane transport” were enriched in the *alvei*-specific core genome; the functional categories “carbohydrate metabolism” and “membrane transport” were enriched in the *paralvei*-specific core genome (Fig. 3a). These species-specific genes indicate the putative niche differentiation between *alvei* and *paralvei*.

Three and two complete pathway modules are present in the *alvei* and *paralvei*-specific core genomes (Fig. 3b), respectively. The EvgS-EvgA two-component regulation system in *Escherichia coli* is a transcriptional regulator of drug efflux genes and closely is related to multi-drug resistance [10]. The cellobiose phosphotransferase system confers the assimilation of cellobiose by cleaving the disaccharide into glucose and glucose-1-phosphate, which serve as carbon and energy sources [11, 12]. MetD (MetI-N-Q) is a high-affinity transport system for methionine, which is an important amino acid involved in numerous metabolic processes in bacteria [13]. The CreC-CreB (carbon source-responsive) two-component regulation system in *Escherichia coli* affects a number of functions, including the intermediary carbon catabolism and intracellular redox state [14]. 2-O-α-mannosyl-D-glycerate is taken up by the MngA phosphotransferase system and utilized as a sole carbon source [15]. Our analysis revealed that genes related to metabolic pathways, antimicrobial resistance and virulence were part of these unique species-specific core genomes, which helped us to characterize the genomic differences between *alvei* and *paralvei*. These species-specific genes might serve as a mark for distinguishing *alvei* and *paralvei*.

### Macromolecular secretion systems reflected the pathogentic potential of *Hafnia* and Mobile genetic elements mediated the genomic plasticity

**Fig. 4:**
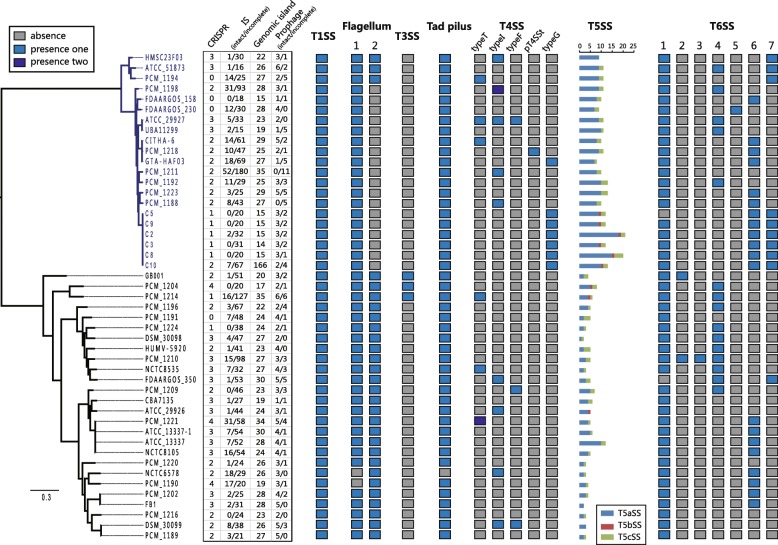
Macromolecular secretion systems distribution in *Hafnia*. A coloured box indicates the presence of a macromolecular system in a genome and a grey box indicates the absence of a macromolecular system

**Fig. 5:**
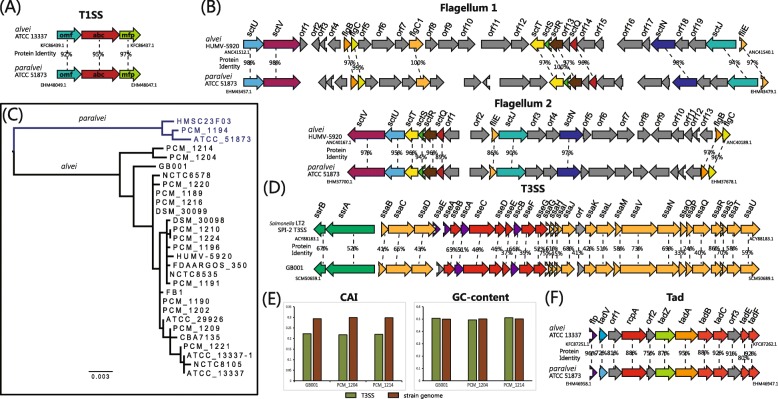
Models of T1SS, flagellum, and T3SS in *Hafnia*. The same genes are shown in the same colour and linked by dotted lines. The percentage protein identities of each homologous gene are shown. **a** The genetic organization of T1SSs. **b** The genetic organizations of the flagellum. **c** ML phylogeny of core protein sequences in the Flagellum 2 cluster. **d** The genetic organization of T3SS. Genes encoding proteins for the apparatus (yellow), effectors and translocators (red), chaperones (purple), and regulators (green) are shown. **e** Comparison of CAI and GC-content between gene clusters of T3SS and coding regions of genomes. **f** The genetic organization of the Tad pilus system

**Fig. 6:**
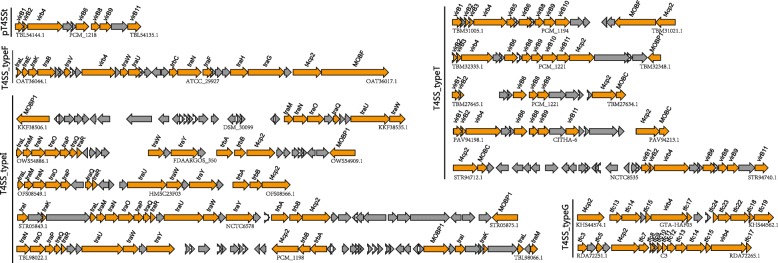
The genetic organization of T4SS in *Hafnia*. The genes in T4SSs are shown in yellow and the grey arrows represent ORFs. The same organization in a subtype was shown using one reference model. In typeF, strains DSM_30099 (shown in Fig. 6), PCM-1209 and ATCC_29927 have identical organization. In typeT, strains PCM_1194 (shown in Fig. 6), PCM_1214, and ATCC_29927 exhibit the same organization. In typeI, strains NCTC6578 (shown in Fig. 6), PCM_1188, PCM_1198, PCM_1211, ATCC_29926, and ATCC_29927 also have similar organization

Secretion is an essential task for bacteria to interact with their surrounding environment [16]. In particular, many virulence factors in pathogens are secreted [17]. The production of extracellular proteins is important for many aspects of bacterial competition and adaptation such as virulence, antimicrobial resistance, detoxification and scavenging [18]. In gram-negative bacteria, six types of secretion systems (T1SS to T6SS) have been identified and well characterized by numerous experimental studies [19–21]. Here, we identify occurrences of the macromolecular secretion systems in 47 *Hafnia* genomes using MacSyFinder [22]. The model and evolution of macromolecular secretion systems identified in *Hafnia* genomes are described in Figs. 4, 5, and 6. In the following sections we describe the models of each type of macromolecular secretion system.

Gene clusters of types I, III, IV, V, VI and homologues of the flagellum, and Tad pilus secretion systems, were found in the *Hafnia* genomes (Fig. 4). Although T1SS, Flagellum 1, Tad pilus, and T6SS-1 are restricted to *Hafnia*, T3SS, T4SS, T5SS, and other T6SSs are not wholly exclusive to strains of *alvei* and/or *paralvei*. These strain-specific secretion systems might be horizontally transferred from other species. Furthermore, numerous mobile genetic elements (MGEs) included clustered regularly interspaced short palindromic repeats (CRISPRs), insertion sequences (ISs), genomic islands, and prophages that have been identified across the study genomes (Fig. 4). These elements are the major contributors to horizontal gene transfer (HGT), and drive the adaptation of bacteria to diverse niches. The diverse secretion systems may be acquired as part of the MGEs.

### Conserved the type I secretion system

T1SS is composed of three indispensable membrane proteins, an ABC transporter (ABC: providing an inner membrane channel), a membrane porin (OMF: forming the outer membrane channel) and an inner membrane anchored adaptor protein (MFP: connecting the OMF and the ABC components) [23]. T1SS can secrete many proteins, including haemolysins for pathogenesis in the host organism, and some extracellular proteases for nutrient acquisition, some bacteriocins for antibacterial activity [18, 20, 24]. We found that the T1SS cluster was present in all 47 *Hafnia* genomes. Both *alvei* and *paralvei* shared a common T1SS cluster (Fig. 5a). The core components ABC, OMF and MFP were encoded together and highly conserved (protein identity > 92, Fig. 5a), thus indicating that this T1SS is restricted and conserved in the *Hafnia* genus.

### Conserved and species-specific flagellum and incidental the type III secretion system

The flagellum and T3SS are two of the most impressively large macromolecular complexes spanning both membranes of gram-negative bacteria [25]. Two types of flagellum systems are identified in *Hafnia* genomes (designated Flagellum 1 and Flagellum 2, Fig. 5c). Flagellum 1 is present in both *alvei* and *paralvei* except for two *alvei* strains (NCTC 6578 and PCM 1190). As shown in Fig. 4, Flagellum 2 is present in all *alvei* strains and only three *paralvei* strains (HMSC23F03,ATCC 51873, and PCM 1194). Furthermore, we performed phylogenetic analysis using the core genes of Flagellum 2 (Fig. 5d), and the resulting tree revealed a similar topology to that of the core genome tree (Fig. 1). Our analysis shows that most strains of *paralvei* do not contain Flagellum 2 due to an earlier deletion event.

T3SS evolved from the flagellum and is at the centre of the export machinery that enables the direct transfer of proteins from the bacterial cytosol into the host cells [25]. T3SSs are usually encoded in a single locus, and many are homologous to components of the flagellar apparatus [26]. Three *alvei* genomes (GB001, PCM 1204, PCM 1214) had a complete set of T3SS genes (Fig. 5d). We compared the CAI (codon adaptation index) and GC-content between the T3SS gene clusters and three host genomes. The T3SS gene clusters displayed an apparent deviation in CAI (Fig. 5e), and it is likely that these T3SS gene clusters were acquired by HGT events. Furthermore we identified the putative homologous T3SS using blastp searches of the NCBI non-redundant protein database. We found that the well-studied T3SS of *Salmonella* pathogenicity island 2 (SPI-2) was closely related to the T3SS of *Hafnia*, and they inhabit similar gene loci and show approximately 54% identity between protein sequences(Fig. 5d). The function of the T3SS encoded by SPI-2 is central to the ability of *S. enterica* to cause systemic infections and for intracellular pathogenesis [27, 28]. It is worth noting that these three *alvei* strains with T3SS have the potential to cause systemic disease.

### Conserved the tight adherence pilus

The Tad pilus secretion system plays a role in biofilm formation, pathogenesis, adhesion or natural transformation in many bacteria [29, 30]. In the *Hafnia* genus, a Tad pilus system has been identified in all but one *alvei* strain (NCTC 6578). The components of the Tad systems of both *alvei* and *paralvei* are encoded within one genetic cluster and share identical genetic organization and high identity of protein sequences (Fig. 5f). Therefore, our results reveal that this Tad pilus system is restricted and conserved in the *Hafnia* genus.

### Strains-specific the type IV secretion system

T4SSs transport a diverse array of substrates, including DNA, DNA-protein complex, and proteins into the host cells, and play fundamental roles in both pathogenesis and adaptation in the host cellular niche. T4SSs are divided into eight subtypes [16, 31]. Our analysis revealed that 23 *Hafnia* genomes possessed T4SSs comprised of types T, I, F, G, and pT4SSt. The strain PCM_1218 possesses pT4SSt, which is a secreted protein system, and other T4SSs in *Hafnia* were the conjugation-related T4SSs. The number of genes in *Hafnia* T4SSs ranges from 7 to 15. As shown in Fig. 6, the genetic organization of T4SSs in *Hafnia* is highly diverse, revealing that the presence of T4SSs is strain-specific but not species-specific. The diversity and strain-specific distribution suggest the likelihood of multiple horizontal transfer events during divergent evolution. As the T4SS phylogenetic analysis generated with shared sequences was not congruent to those obtained with genomic sequences, the distribution of *Hafnia* T4SS phylogenetic clusters within the SecReT4 database [32] was not assessed further.

### Diverse the type V secretion system

T5SS is the simplest and most widespread type of secretion pathways [33]. T5SS encodes the translocator and the passenger domains in a single gene or two partner genes [34, 35]. In this study, based on TXSScan software, T5SSs are divided into three types, designated T5aSS (the classical autotransporter), T5bSS (two partner system), and T5cSS (the trimeric autotransporter) [16]. We found that all 47 *Hafnia* genomes contain T5SSs (Fig. 4). A total of 370 T5SSs were identified, including 285 T5aSSs, 9 T5bSSs, and 76 T5cSSs. Most of the *Hafnia* genome contained T5aSSs and T5cSSs. Only 9 strains including 3 *alvei* and 6 *paralvei* contained one T5bSS. It is interesting to note that there are notable differences in the numbers of T5aSSs between *alvei* (3.3 ± 1.8 per genome) and *paralvei* (9.5 ± 2.5 per genome) (Fig. 4). In consideration of T5aSSs can function as enzymes, adhesins, cytotoxins, or mediate bacterial motility, this differential number of T5SSs suggests that the *alvei* and *paralvei* differ in their adhesion and evasion abilities [36]. The lager number of T5aSSs may play an important role in the pathogenesis of *paralvei*, such as the colonization of host cells, biofilm formation, or evasion of the immune system [36].

### Conversed and diverse the type VI secretion system

**Fig. 7:**
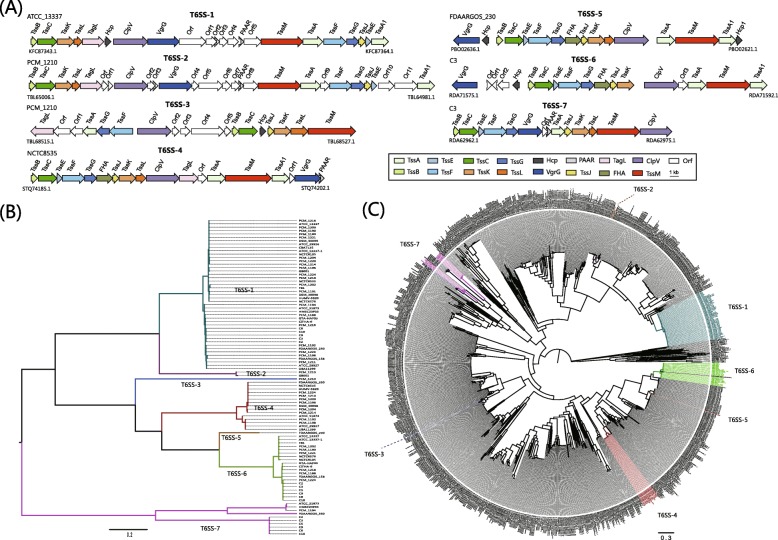
Model and phylogeny of T6SS in *Hafnia*. **a** The genetic organizations of T6SS. The same genes are shown the same colour. **b** ML phylogeny generated from the shared TssK protein sequences in the *Hafnia* T6SS. **c** ML phylogeny generated from the shared TssK protein sequences in the SecReT6 database [41] in combination with the *Hafnia* T6SS data. The different subtypes of *Hafnia* T6SSs are shown in different colours

T6SS is present only in gram-negative bacteria and is a phage-tai-spike-like injection machinery [37]. It is thought to contribute for bacterial pathogenesis by the translocation of substrates to the host cells and competition with other bacteria in their niches [38, 39]. T6SS has not been reported previously in *Hafnia*. In this study, all *Hafnia* genomes demonstrated the possession of one or more T6SSs (Fig. 4), suggesting that they may confer some benefit in terms of host colonization and/or pathogenic potential. Although previous studies suggest that strains carrying T6SS may selectively target proteobacterial commensals for the sake of competitive advantage over other potential competitors [40], the function of T6SS in *Hafnia* remains to be elucidated with further experiments. Based on genetic organizations and homology, the T6SS in *Hafnia* genomes were divided into 7 subtypes (Fig. 7a). T6SS-1 was conserved in most *Hafnia* strains, but absent in strains C5 and FDAARGOS_350. Nevertheless, similar to T4SS, the presence of T6SS-2 to − 7 was strain-specific but not species-specific. The presence of diverse T4SSs and T6SSs further confirms that HGT is a normal event that occurs in *Hafnia*.

We constructed an ML tree based on the TssK protein sequences to span the diversity present in *Hafnia* T6SS (Fig. 7b). In the ML tree, the strains with T6SSs of the same subtype T6SSs form a unique cluster; that is distinct from other subtypes. A similar ML tree generated from TssF is provided in Additional file 5: Figure S1A. This observation reveals the diversity of *Hafnia* T6SS and the possibility of multiple evolutionary origins. To intuitively understand the diverse origins from an overall perspective, we performed phylogenetic analysis using the shared TssF proteins in the SecReT6 database [41] in combination with the *Hafnia* T6SS data. As shown in Fig. 7c, the distinct *Hafnia* T6SSs scatter in different locations, showing high homology to other T6SSs from other species, such as *Escherichia coli*, *Salmonella enterica*, *Photorhabdus asymbiotica, Enterobacter asburiae*, and *Citrobacter rodentium*. The phylogenetic trees created from the shared TssFs exhibited similar topologies (Additional file 5: Figure S1B). The data indicate that distinct subtypes of *Hafnia* T6SSs might be horizontally transferred from diverse donor species.

### Virulence genotypic profile revealed the pathogenicity of *Hafnia*

**Fig. 8:**
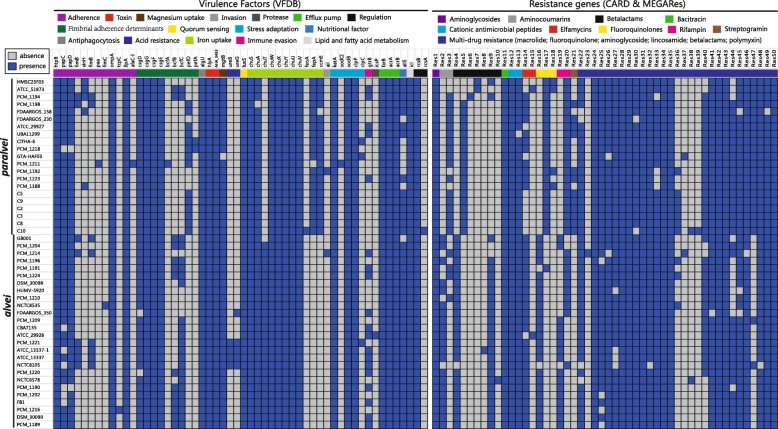
The heatmap of the distribution of virulence factors and resistance genes across all 47 genomes. Blue color represents the presence of a gene, and grey represents its absence. In the current presentation, we removed the virulence factors of the previously studied macromolecular secretion system and O-antigen/LPS/capsule

Mobilome-based elements, such as virulence factors and resistance genes, are very important in pathogenicity and inter-strain variation. In our study, all 47 *Hafnia* genomes were also locally aligned against the VFDB database, MEGARes, and CARD database to detect virulence factors and resistance genes [42–44]. Virulence factor analysis of the strains examined in this study found that virulence factors represented the greatest numbers of genes among the *Hafnia* strains (214 virulence factors, Additional file 6: Table S5). Except for previously studied genes related to the macromolecular secretion system, the major virulence factors identified in all strains were associated with adherence (*ompA*, *ilpA*, *papD*, *papC*, *htpB*, and *csg* fimbriae), toxin (*hlyA* and AHA_3493), iron uptake (*chu* operon), stress adaptation (*katA, clpP,* and *sodB*), and efflux pump (*farB* and *acrAB*). Additionally, *algU* (antiphagocytosis), *mgtB* (magnesium uptake), *luxS* (quorum sensing), *allS* (nutritional factor), *icl* (lipid and fatty acid metabolism) and *rcsB* (regulation) were identified as common *Hafnia* virulence factors (Fig. 8).

Several virulence genes were identified as species-specific virulence factors that occurred in the majority of strains of one species but were found infrequently in strains of the other species, making them good discriminators between *alvei* and *paralvei* (Fig. 8). The *alvei*-specific genes included *bcfC*, and *chuT*. The *chu* operon except for *alvei*-specific *chuT* was found to be prevalent in *Hafnia* strains and was termed the haem transport locus, which appears to be widely distributed among pathogenic *E. coli* strains [45]. The *paralvei*-specific genes identified were associated with fimbrial adherence (*pefD*) and iron uptake (*ccmE* and *fepA*). This differential distribution of virulence factors suggests that *alvei* and *paralvei* differ in their adhesion abilities and iron absorption capacities.

### Antimicrobial genotypic and phenotypic profiles in *Hafnia*

Fifty resistance genes associated with nine different classes were identified (Fig. 8, Additional file 7: Table S6). All *Hafnia* genomes contained multiple resistance genes related to aminoglycoside, beta-lactam, bacitracin, cationic antimicrobial peptide, fluoroquinolone, and rifampin. We observed the diversity of *acc* gene alleles in *Hafnia* genomes (Fig. 8, Additional file 7: Table S6). Except NCTC8105, all *alvei* strains contained *acc-3* allele. Instead, *paralvei* strains contained *acc-1* (18/21), *acc-5* (1/21), and *acc-2* (2/21) alleles. This observation was in agreement with previous study [46]. Similarly, the *gyrB* alleles related to fluoroquinolone also showed species-specific divergence (Res16 and Res17, Additional file 7: Table S6). Except error location of PCM_1191, the ML tree based on *gyrB* gene sequences separated *Hafnia* into two species clades with similar topology to core genome tree (Additional file 8: Figure S2). Additionally, we found that many genes encoding efflux pump related to multi-drug resistance are prevalently present in the genome of all *Hafnia* strains.

All of the 20 *Hafnia* strains were tested for susceptibility against 21 antimicrobial agents; the results are listed in Additional file 9: Table S7. All strains were uniformly susceptible to ticarcillin-clavulanic acid, cefoperazone-sulbactam, cefepime, imipenem, amikacin, tobramycin, ciprofloxacin, levofloxacin, tigecycline, and trimethoprim-sulfamethoxazole (Additional file 9: Table S7). Almost all strains showed prevalent resistance to amocicillin-clavulanic acid (100%; *n* = 20) and colistin (90%; *n* = 18). Meanwhile, we observed partial resistance against piperacillin-tazobactam (25%; *n* = 5), ceftriaxone (35%; *n* = 7), ceftazidime (35%; *n* = 7), and ertapenem (20%; *n* = 4). In terms of species susceptibility pattern, some *alvei* strains were resistant to aztreonam (2/13), meropenem (4/13), and chloramphenicol (2/13), meanwhile, one *paralvei* strain (PCM_1198) was resistant to doxycycline and minocycline.

## Discussion

*Hafnia* genus belongs to the family *Enterobacteriaceae*, and was isolated from the faces of mammals, birds, reptiles, and fish, as well as from soil, water, and foods [47]. It is an opportunistic pathogens that has been implicated in both nosocomial and community-acquired infection. Although it was first identified in 1954, Its taxonomy has remained an unsolved riddle. It is also unclear what the distribution and frequency of these two species are in clinical specimens. Furthermore, the pathogenesis and virulence-related genotype are still not clear. Improvements in next-generation sequencing have resulted in an upsurge of bacterial genome sequences and bioinformatics tools. They have been useful for bacterial taxonomy and provide the opportunity to determine the divergence between species [48]. The genome sequence data of 20 *Hafnia* strains generated in this study in combined with all 27 publicly available assemblies represented an important contribution to the broader goal of using whole-genome sequences for comparative studies of *Hafnia*. In this study, phylogenetic and population genetic analysis based on core genomes in combination with whole-genome average nucleotide identity exhibited a reliable delineation of genetic relationships between *alvei* and *paralvei* and provided a high-resolution taxonomy of *Hafnia*. We also identified one previously misclassified as *alvei* genome, ATCC 51873, which should be labeled as paralvei.

Bacterial genomes are much more variegated than those of eukaryotes. The diverse niches led to the inter-species variations and diverse pathogenesis in strains that could be due to horizontal gene transfer. The diversity of the *Hafnia* strains is evidenced in the fact that the pan-genome of *alvei* and *paralvei* are open. Bared on the COG analysis, both *alvei* and *paralvei* contained a larger proportion of core gene families involved in the transcription (category K), energy production (category C), transport and metabolism of carbohydrates and amino acids (categories G and E). The diverse niche of *Hafnia* may specifically shape its genome characteristics during ecological adaptation and divergent evolution. Thus, we determined the species-specific core genomes of *alvei* and *paralvei* separately. In the KEGG annotation, metabolism and membrane transport were the major functional annotations of these two species-specific core genomes. We found three complete pathway modules included the EvgS-EvgA two-component regulation system, methionine transport system, and cellobiose PTS system, are present in the *alvei*-specific core genomes. The *paralvei*–specific core genomes included the CreC-CreB two-component regulation system and 2-O-α-mannosyl-D-glycerate PTS system. Therefore, the species-specific metabolism-associated gene profiles for *alvei* and *paralvei* may reflect the specific nutrient niches in the different host and environments. Moreover, the presence/absence of these species-specific genetic elements would appear to provide a simple and fast approach for identifying *alvei* and *paralvei* using PCR.

The *Hafnia* genomes also harbour the numerous MGEs, and diverse virulence-related genetic profiles that can result from HGT. In particular, the presence of numerous MGEs included CRISPRs, ISs, genomic islands, and prophages may promote an increase in the rate of HGT thus enhancing inter-species divergence. The elements acquired by HGT include metabolic pathways, fitness factors, virulence factors, and resistance genes and can confer fitness, competition, virulence, and antibiotic resistance on the receptor strains [49]. Our observations highlight the potential of *Hafnia* to engage in robust HGT, which can promote the adaptation of *Hafnia* to diverse niches and the acquisition of pathogenicity.

Our comparative genomic analysis found that macromolecular secretion systems are widely distributed in the *Hafnia* genus. These systems include T1SS, flagellum, T3SS, Tad pilus, T4SS, T5SS, and T6SS. Our study revealed the diversity in T4SSs, T5SSs, and T6SSs and the conservation of T1SS, Flagellum 1, and Tad pilus among all strains. It is worth noting that three *alvei* strains acquired a putative SPI-2 related T3SS from *Salmonella enterica* by HGT event. The observation of this case of pathogenic evolution indicates the potential of the strain to cause severe systemic infection. In addition to possessing a macromolecular secretion system within their genomes, *Hafnia* strains normally harbour many virulence factors that have been experimentally verified to be important for bacterial pathogenicity [42]. These include the secretion of toxic substances, the production of adherence factors that mediate adhesion and interaction with host cells, metal adsorption, and antibiotic resistance, indicating the ability of *Hafnia* to damage intestinal epithelial barriers, invade host cells, cause systemic infection, and exhibit multiple drug resistance. Antimicrobial genotypic and phenotypic profiles were also investigated in this study. All *Hafnia* strains contained several resistance genes related to multiple antimicrobial resistance. Additionally, we found many genes encoding efflux pumps in both *alvei* and *paralvei*. These results imply the possibility that the *Hafnia* strains have acquired multiple resistances to multiple antimicrobials. The susceptibility profile of *Hafnia* in this study highlighted prevalent resistance to Amocicillin-clavulanic acid and Colistin, partial resistance to Piperacillin-tazobactam, Ceftriaxone, Ceftazidime, and Ertapenem. This finding provides important clues for clinical treatment and antibiotic use.

Our comparative analysis also showed differences in macromolecular systems, virulence, and resistance profiles between *alvei* and *paralvei*. The *alvei*-specific virulence genes included *bcfC* (fimbrial adherence), and *chuT* (iron uptake). Most strains of *paralvei* do not contain Flagellum 2 as a result of an earlier deletion event. The strains of paralvei hold a larger number of T5sSSs, *pefD* (fimbrial adherence), *ccmE* and *fepA* (iron uptake). These variations of virulence-related profiles suggests the divergence in adhesion abilities and iron absorption capacities. The alleles of resistance genes, *aac* and *gyrB* also provided a simple method for identification of both species. We also found that closely related strains of the same species also show genomic diversity. The T3SSs, T4SSs, T5SSs, T6SSs, virulence factors, and resistance genes in *Hafnia* exhibited high diversity and strain-specific distribution, indicating the likelihood of multiple horizontal transfer events from diverse donor species during divergent evolution. These strain-specific variations indicates that *Hafnia* strains vary in their pathogenesis.

## Conclusion

In this study, application of whole-genome based phylogeny, population genetic analysis, and ANI analysis provided precise insights into taxonomic relationships among the *Hafnia* genus. The genome data presented in this study offered important genetic information and a framework for further research. We determined the species-specific genetic profiles that were associated with metabolism, membrane transport, virulence, and antimicrobial resistance, indicating the putative differentiation in niche and pathogenicity between *alvei* and *paralvei*. These species-specific genes might serve as a mark for distinguishing *alvei* and *paralvei*. *Hafnia* is an opportunistic pathogen that has been associated with several diseases, including gastroenteritis, bactereamia, and respiratory tract infection. Conserved T1SSs, Flagellum, and Tad pilus and diverse T4SSs, T5SSs, T6SSs, virulence genes, and resistance genes were found in *Hafnia* pan-genome. We constructed this extensive genomic evaluation of the virulence-related genetic profiles of *Hafnia* that can help us comprehend the pathogenesis of *Hafnia*. Clinicians should be aware of this organism and pay attention to *Hafnia* because it possesses many genetic elements related to virulence and can cause clinically significant infection in an appropriate host.

## Methods

### Bacterial isolates, DNA extraction

Our 20 *Hafnia* strains including 7 *paralvei* and 13 *alvei* were obtained from the Polish Collection of Microorganisms (PCM) at the Hirszfeld Institute of Immunology and Experimental Therapy, Polish Academy of Sciences (Wroclaw, Poland). All of these strains were found to have different lipopolysaccharides using immunochemical methods. The genetic diversity of O-antigens in our 20 strains has been reported [50]. These strains were grown in liquid medium as previous described [50] and then harvested using centrifugation (1380 *xg* for 15 min at 4 °C). Bacteria Extraction Kit (CWBIO Co., Ltd., China) were used for DNA extraction from each isolate according to the manufacturer’s instructions.

The whole genome of PCM 1220 was sequenced using Pac Bio RS II (Pacific Biosciences), with a depth of approximately 100-fold coverage. The other 19 strains were sequenced using Solexa pair-end sequencing technology (Illumina, Little Chesterford, Essex), with a depth of 90 to 100 fold coverage. For PCM1189 and PCM 1220, a 20-kb library was constructed and end-repaired, and the adaptors were then ligated to generate Single Molecule Real Time (SMRT) bells for circular consensus sequencing. The reads produced with the Pac Bio RS II were de novo assembled using MaSuRCA [51, 52], and those produced with the Solexa pair-end sequencing technology were de novo assembled using Velvet Optimiser v2.2 [53]. The annotation of the genome sequence was conducted using the NCBI Prokaryotic Genome Annotation Pipeline (http://www.ncbi.nlm.nih.gov/genome/annotation_prok). The nucleotide sequences and statistics were submitted to NCBI GenBank (accession numbers listed in Tables). In addition, 27 publicly available *Hafnia* genomes were obtained from NCBI GenBank. All strains used in this study are presented in Additional file 1: Table S1.

### Identification of gene orthologous group

OrthoFinder [54] was used to determine orthologous families of the pan-genome. All protein sequences were compared using a BLASTp all-against-all search with an E-evalue cutoff of <1e-3. The single-copy core gene, pan gene families and core genome families were extracted from the OrthoFinder output file. Nucleotide sequences of single-copy core genes were extracted according to protein ID.

### Phylogenetic analysis

According to the identification of gene orthologous clusters, a total of 2045 single-copy orthologous core genes shared by per genome. To determine the single nucleotide polymorphisms (SNPs), the nucleotide sequences of single-copy core genes using in core genome phylogenetic analysis were aligned using MAFFT [55] with the default parameter. The SNPs were integrated according to the arrangement of the single-copy genes in complete genome *alvei* FB1. The phylogeny of SNPs were inferred using the Maximum Likelihood (ML) algorithm in PhyML [56], [with the GTR model of nucleotide substitution and c-distributed rates among sites]. FigTree 1.4.3 (http://tree.bio.ed.ac.uk/software/figtree) was employed to show the trees. In consideration that homologous recombination caused by horizontal gene transfer occurring in bacterial populations and can confound phylogenetic analysis. We identified and removed putative recombinational regions of the set of SNPs of single-copy core genes, using CloneFrameML [57]. The ML tree of Flagellum 2, TssK, and TssF generated protein sequences which were aligned using the default parameter of MAFFT [55], and were constructed by PhyML using the WAG amino acid substitution model of evolution [56].

### Population structure analysis

The population genetic structure of the *Hafnia* genus was inferred using the software STRUCTURE 2.3.4 [7] based on SNPs of single-copy core genes, [with k (the number of subpopulation 1–10) and Rep (repeats 5)]. STRUCTURE Harvester [58] assumed *k* = 2 subpopulations and correlated allele frequencies, linkage model based on maker distances in base pairs, 10,000-iteration burnin and 10,000 iterations of sampling.

### Whole-genome nucleotide identity

The average nucleotide identity (ANI) and tetramer usage pattern were calculated for the 47 genome dataset using JSpecies 1.2.1 [8], using default parameters. The result was visualized using the pheatmap R packages.

### Pan-genome analysis

The pan-genome analysis were separately performed on two dataset of 21 *paralvei* and 26 *alvei* genomes. According to the Heap’s law pan-genome model described in reference [9], the total number of gene clusters is shown for increasing values of the number *N* of genomes. The curve was a least squares fit of the power law *n* = *кN*^*γ*^ to averages. *N* is the number of genomes, *n* is the number of core gene clusters, and *к* and *γ* parameters. The exponent *γ* > 0 indicates an open pan-genome species.

### Species-specific core genome comparison

To construct accessory genome, we excluded the core gene families and low frequency gene families (shared by less than 10 strain genomes) from pan-genome. To examine the accessory-genome in more detail, we constructed a cluster map for the gene families across all 47 genomes using the heatmap clustering command from the pheatmap R packages (Fig. 2d and Additional file 10). We termed the results as “the species-specific core genome” (Additional file 4: Table S4), which represents the set of gene families that are shared across all strains of a species.

### Functional category

We inferred the functional category of the core gene families of *alvei* and *paralvei* using the Cluster of Orthologous Group (COG) assignment [59]. The functional annotation of proteins was performed by alignment against the COG database of NCBI using amino acid sequences. Functional analysis of species-specific core genome was done according to the KEGG database.

### Identification of MGEs

The clustered regularly interspaced short palindromic repeats (CRISPRs) were predicted with the CRISPR recognition tool (CRT1.2) [60] with default parameters. Insertion sequences (ISs) were predicted using the IS Finder database. Genomic islands were predicted using IslandViewer. The PHAge search tool (PHAST) was utilized to find the prophages.

### Identification of macromolecular secretion systems

The detection and visualization of Macromolecular systems in *Hafnia* genus were performed using the program MacSyFinder [22] and TXSScan [16] on the default parameters (http://mobyle.pasteur.fr/cgi-bin/portal.py#forms::txsscan.). Furthermore, the T4SS and T6SS were also predicted using SecReT4 [32] and SecReT6 [41] on the default parameters, respectively, which annotates and locates the components of T4SS and T6SS on the genome sequences.

### The calculation of codon usage and GC-content

We use CodonW software (available at http://sourceforge.net/projects/codonw) computing statistical parameters of nucleotide composition of gene clusters and genomes such as GC-content and CAI (Codon adaptation index).

### Identification of virulence factors and resistance genes

To identify the virulence factors and resistance genes, the protein sequences of all 47 genomes were aligned using blastp with an E-value cutoff of <1e-6, identity > 50%, and coverage > 60% against the dataset from Virulence Factors Database (VFDB), MEGARes, and Comprehensive Antibiotic Resistance Database (CARD) [42–44]. In the final presentation, we removed the virulence factors of previous studied macromolecular secretion system and O-antigen/LPS/Capsule (Fig. 7). The heatmap of distribution of virulence factors and resistance genes were generated by the pheatmap R packages.

### Antimicrobial susceptibility testing

Antimicrobial susceptibility testing was performed for 21 antibiotics, including amocicillin-clavulanic acid, ticarcillin-clavulanic acid, piperacillin-tazobactam, ceftriaxone, ceftazidime, cefoperazone-sulbactam, cefepime, aztreonam, imipenem, meropenem, ertapenem, amikacin, tobramycin, ciprofloxacin, levofloxacin, doxycycline, minocycline, tigecycline, colistin, trimethoprim-sulfamethoxazole, and chloramphenicol. MICs were determined by the broth microdilution using dehydrated panels AST-N334 and AST-N335 (Biomerieux, Vitek2) according to standard protocols. Resistance was defined using CLSI criteria. Susceptibility data for our 20 *Hafnia* strains are shown in Additional file 9: Table S7.

## Supplementary information

**Additional file 1: Table S1.** Genetic characteristics of strains in the current research.

**Additional file 2: Table S2.** List of 2045 single-copy genes shared by 47 *Hafnia* strains.

**Additional file 3: Table S3.** Average nucleotide identity (ANI)(%) based on whole genome alignments. ANI values in the species *paralvei* are in red.

**Additional file 4: Table S4.** List of *alvei* and *paralvei*-specific core genomes with KEGG annotation.

**Additional file 5: Figure S2.** Maximum likelihood phylogenetic trees generated from the TssF protein sequences in *Hafnia* T6SS (A) in combination with the SecReT6 database (B).

**Additional file 6: Table S5.** List of virulence factors in the VFDB database that were identified in *Hafnia* strains.

**Additional file 7: Table S6.** List of resistance genes in the CARD and MEGARes database were identified in *Hafnia* strains.

**Additional file 8: Figure S2.** Maximum likelihood phylogenetic tree generated from the *gyrB* gene sequences in *Hafnia* strains.

**Additional file 9: Table S7.** Antimicrobial susceptibility profiles of *Hafnia*.

**Additional file 10.** The R script command of heatmap clustering for accessory genome analysis.

## Data Availability

The genome sequencing data of our 20 *Hafnia* strains has been deposited at NCBI GenBank under the BioProject accession no. PRJNA523071 and the genome BioSample accession no. (SAMN10963441 - SAMN10963460). All genome sequences and annotations referenced in the manuscript are publically available on the GenBank Database at the accession number provided (Additional file 1: Tale S1).

## Abbreviations

ANI: Average nucleotide identity
BAPS: Bayesian Analysis of Population Structure
CAI: Codon adaptation index
COG: Cluster of Orthologous Group
CRISPR: Clustered regularly interspaced short palindromic repeats
CRISPR: The clustered regularly interspaced short palindromic repeats
*H.**alvei*: *Hafnia alvei*
*H.**paralvei*: *Hafnia paralvei*
HGT: Horizontal gene transfer
IS: Insertion sequence
KEGG: Kyoto Encyclopedia of Genes and Genomes
MGEs: Mobile genetic elements
ML: Maximum Likelihood
NCBI: National Center of Biotechnology Information
ORF: Open reading frame
PHAST: PHAge search tool
T1SS-T6SS: Type I - VI secretion
Tad: Tight adherence
VFDB: Virulence Factors Database
