# Supplementary figures and images for: Comparative genomic analysis of the Hafnia genus reveals an explicit evolutionary relationship between the species alvei and paralvei and provides insights into pathogenicity
Source: BMC Genomics. 2019 Oct 23;20:768. doi: 10.1186/s12864-019-6123-1 (PMC6806506; doi:10.1186/s12864-019-6123-1)

( A )

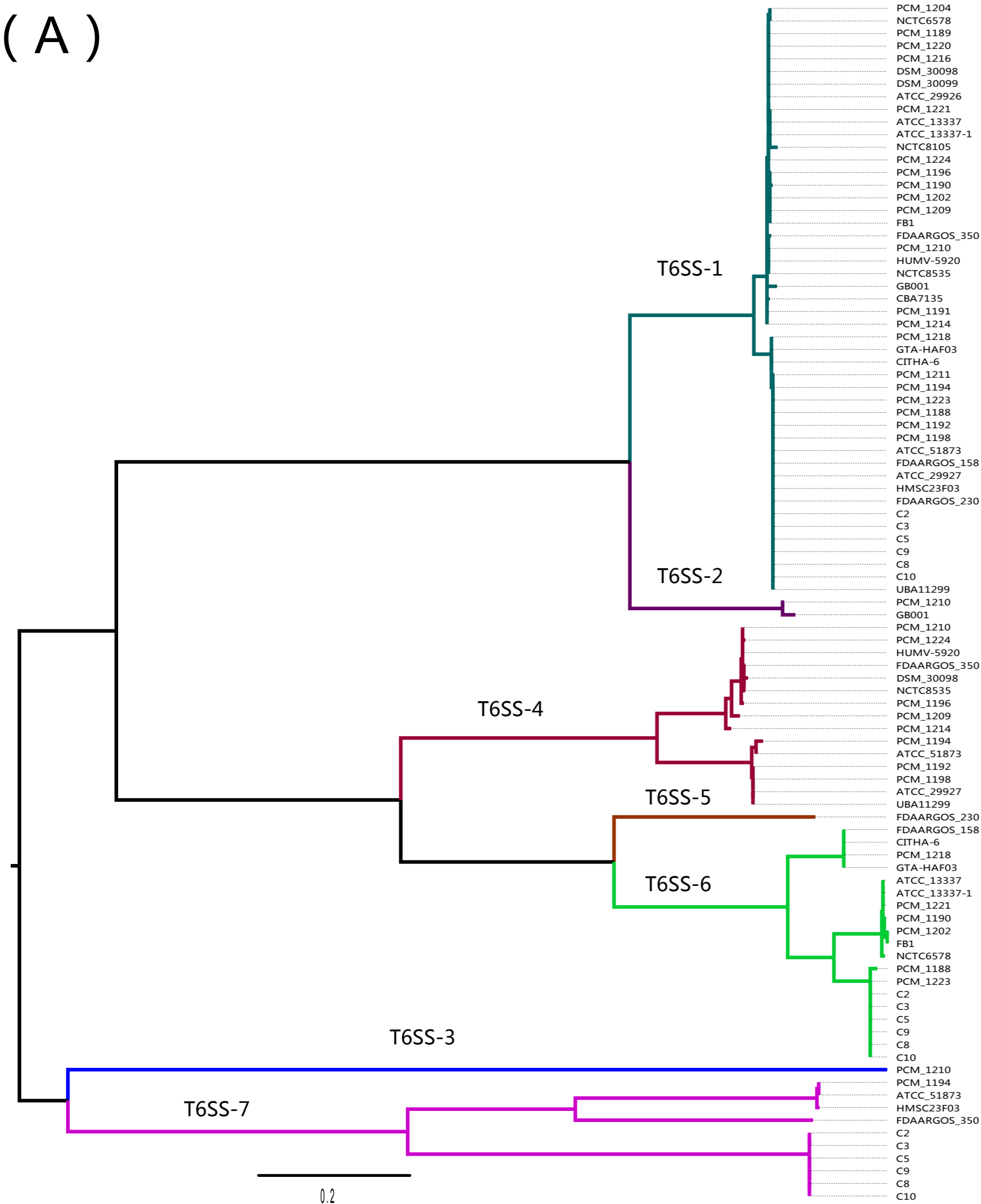

( B )

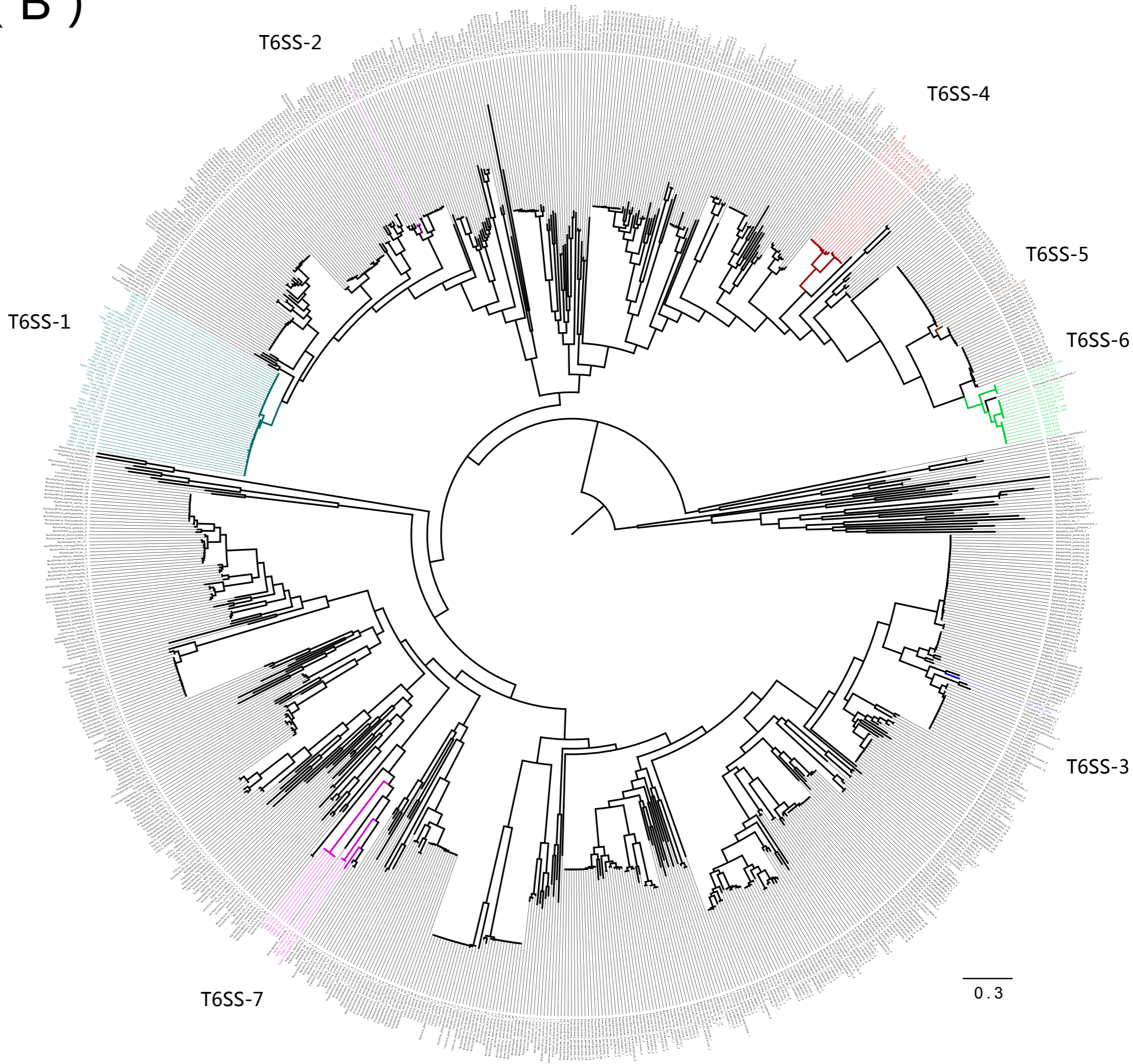

Supplement: Supplementary file 5 — Additional file 5: Figure S2. Maximum likelihood phylogenetic trees generated from the TssF protein sequences in Hafnia T6SS (A) in combination with the SecReT6 database (B). [file 12864_2019_6123_MOESM5_ESM.pdf]

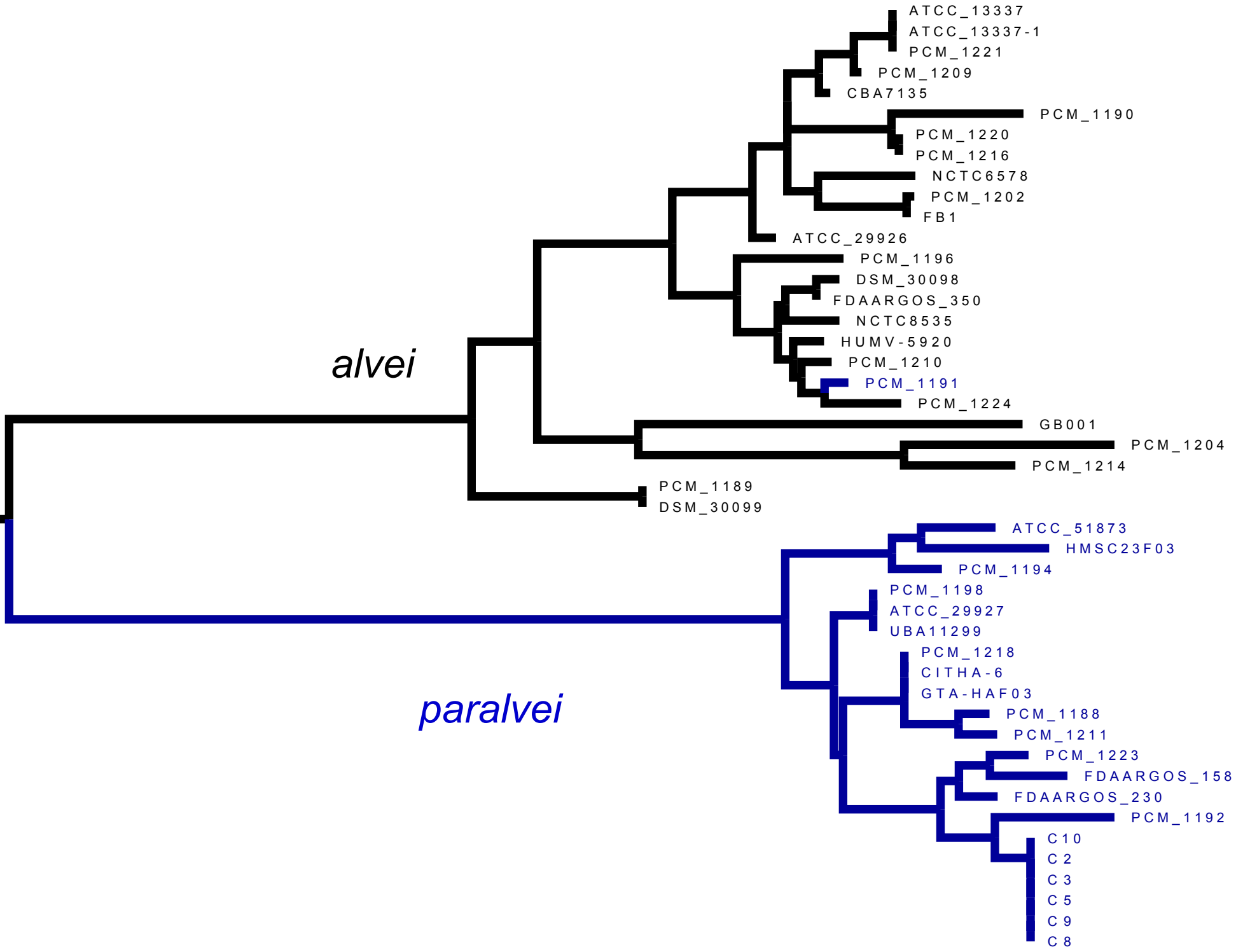

Supplement: Supplementary file 8 — Additional file 8: Figure S2. Maximum likelihood phylogenetic tree generated from the gyrB gene sequences in Hafnia strains. [file 12864_2019_6123_MOESM8_ESM.pdf]
