# Supplementary material for: Comparative genomic analysis of the Hafnia genus reveals an explicit evolutionary relationship between the species alvei and paralvei and provides insights into pathogenicity
Source: BMC Genomics. 2019 Oct 23;20:768. doi: 10.1186/s12864-019-6123-1 (PMC6806506; doi:10.1186/s12864-019-6123-1)
Supplement: Supplementary file 10 — Additional file 10. The R script command of heatmap clustering for accessory genome analysis. [file 12864_2019_6123_MOESM10_ESM.docx]

The R pheatmap package was downloaded from pheatmap (https://github.com/raivokolde/pheatmap).

The input file: accessory_blast_out.txt was the blast result of accessory genome to strain genomes.

The output file: accessory_clustering_heatmap.pdf was Fig. 2d. The species-specific core genes was extracted from the output file: accessory_neworder.txt.

- setwd(‘/home/Hafnia/accessory_clustering/’)
- library(pheatmap)
- a <- read.table(‘accessory_blast_out.txt’,header = T)
- rownames(a) <- a[,1]
- b <- a[,-1]
- b <- as.matrix(b)
- pdf(‘/homw/Hafnia/accessory_clustering/accessory_clustering_heatmap.pdf’,family=’GB1’)
- pheatmap(b,cluster_cols=T,cluster_rows=F,color=colorRampPalette(c("grey",'blue'))(1000), fontsize_row=8, fontsize_col=10, border_color=F, legend=T)
- list=pheatmap(b, cluster_cols=T, cluster_rows=F)
- order_col = list$tree_col$order
- datat = data.frame(b[,order_col])
- datat = data.frame(rownames(datat),datat,check.names =F)
- write.table(datat,file="accessory_neworder.txt",row.names=FALSE,quote = FALSE,sep='\t')
- dev.off()
